# Mediterranean Diet as a Shield against Male Infertility and Cancer Risk Induced by Environmental Pollutants: A Focus on Flavonoids

**DOI:** 10.3390/ijms23031568

**Published:** 2022-01-29

**Authors:** Luigi Montano, Alessandro Maugeri, Maria Grazia Volpe, Salvatore Micali, Vincenzo Mirone, Alberto Mantovani, Michele Navarra, Marina Piscopo

**Affiliations:** 1 Andrology Unit and Service of Lifestyle Medicine in UroAndrology, Local Health Authority (ASL), 84124 Salerno, Italy; l.montano@aslsalerno.it; 2 PhD Program in Evolutionary Biology and Ecology, University of Rome “Tor Vergata”, 00133 Rome, Italy; 3 Department of Chemical, Biological, Pharmaceutical and Environmental Sciences, University of Messina, 98166 Messina, Italy; amaugeri@unime.it; 4 Institute of Food Sciences, National Research Council, CNR, 83100 Avellino, Italy; mariagrazia.volpe@isa.cnr.it; 5 Urology Department, University of Modena and Reggio Emilia, 41124 Modena, Italy; salvatore.micali@unimore.it; 6 Department of Neurosciences, Science of Reproduction and Odontostomatology, University of Naples Federico II, 80126 Naples, Italy; mirone@unina.it; 7 Department of Food, Safety, Nutrition and Veterinary public health, Italian National Health Institute, 00161 Roma, Italy; alberto.mantovani@iss.it; 8 Department of Biology, University of Naples Federico II, 80126 Napoli, Italy; marina.piscopo@unina.it

**Keywords:** pollution, flavonoids, cancer, male infertility, Mediterranean diet, heavy metals, bisphenols, polycyclic aromatic hydrocarbons, dioxins, phthalates

## Abstract

The role of environmental factors in influencing health status is well documented. Heavy metals, polycyclic aromatic hydrocarbons (PAHs), polychlorinated biphenyls, dioxins, pesticides, ultrafine particles, produced by human activities put a strain on the body’s entire defense system. Therefore, together with public health measures, evidence-based individual resilience measures are necessary to mitigate cancer risk under environmental stress and to prevent reproductive dysfunction and non-communicable diseases; this is especially relevant for workers occupationally exposed to pollutants and/or populations residing in highly polluted areas. The Mediterranean diet is characterized by a high intake of fruits and vegetables rich in flavonoids, that can promote the elimination of pollutants in tissues and fluids and/or mitigate their effects through different mechanisms. In this review, we collected evidence from pre-clinical and clinical studies showing that the impairment of male fertility and gonadal development, as well as cancers of reproductive system, due to the exposure of organic and inorganic pollutants, may be counteracted by flavonoids.

## 1. Introduction

**Figure 1:**
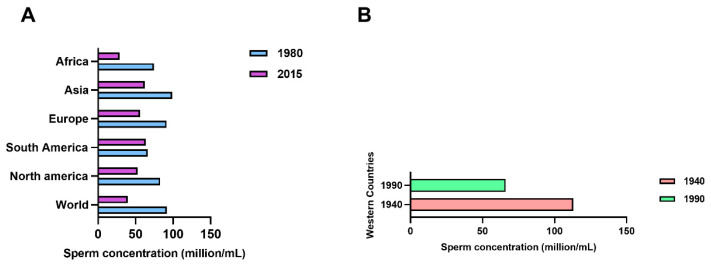
Decrease rates of sperm concentration worldwide. Sperm concentration declines in several areas of the world from 1980 to 2015 (**A**), and from 1940 to 1990 (**B**).

In recent decades, the role of environmental factors in influencing population health has become increasingly evident, so that chemical and physical pollutants are now considered the most important public health threat, whose effects, including transgenerational ones, come to be well reported in the scientific literature [1]. The World Health Organization (WHO) estimates that around a quarter of diseases are due to prolonged exposure to environmental pollutants [2] and air pollution is among the first risk factors for cardiovascular, chronic degenerative diseases, premature deaths and reproductive dysfunctions [3,4]. Noticeably, many outdoor and indoor pollutants carry out their action as endocrine disruptors [5] altering inter-cellular signaling and also inducing oxidative stress [6]. Excess of radical oxygen species (ROS) not balanced by the presence of reductive activity represents a central molecular mechanism of damage for various macromolecules (DNA, proteins, lipids) which, if not properly repaired, can cause inflammatory processes up to neoplastic transformation [7]. Indeed, the imbalance of antioxidant defenses and detoxification processes provides a logical explanation for the occurrence of oxidative stress-related diseases in humans [8]; including the increased susceptibility to pollutants [9]. The reproductive system appears to be particularly sensitive to environmental stresses and an increasing amount of in vitro and in vivo toxicological studies are performed on this system. In fact, besides its obvious functional importance, the reproductive system can also represent a sentinel to environmental stresses; the epidemiological and clinical data available today, in particular, on male infertility, seem to confirm this sensitivity [10,11]. A strong trend of decline, already described for sperm concentration from 113 × 10^6^/mL in 1940 to 66 × 10^6^/mL in 1990 [12], is observed for testosterone levels [13]. Sperm decline has been also reported in Africa, China, India, Brasil from 1980 to 2015 [14,15] (Figure 1).

The incidence of male infertility has increased steadily in many countries [16,17]. The changes in sperm production correspond precisely to the increased introduction of chemicals in the living environment, especially after 1940, and were initially thought to be due solely to maternal exposure to environmental estrogens [18]; based on the current knowledge, especially from experimental data, these effects appear to be due to different types of endocrine disrupting chemicals (EDCs) [18]. EDCs can act through several mechanisms, targeting either nuclear receptors for steroid hormones (i.e., estrogen and androgen receptors), other nuclear receptors (i.e., peroxisome proliferator-activated receptors), steroidogenic enzymatic cascades, FSH/LH balance and at central and/or peripheric levels. Noticeably, the developmental phase is most sensitive: EDC exposure is involved the “testicular dysgenesis syndrome” with poor semen quality and increased risk of congenital anomalies such as hypospadias and cryptorchidism as well as of testicular cancer. Adult exposure to EDC (e.g., in highly polluted environments) may also affect spermatogenesis and male fertility. Also, EDC exposure might synergize with other conditions inducing impaired sperm production, including systemic diseases and genetic factors such as karyotype anomalies and Y chromosome microdeletions [18,19,20]. Indeed, the extensive use of EDCs appears to be involved not only in the fall in sperm count but also in the increased incidence of hypospadias, cryptorchidism and testicular malignancies [20]. The incidence of primary testicular tumors (TC), especially testicular seminomas has increased worldwide and the percentage over the past decades has risen approximately 1.2% per year [21,22,23]. TC generally affects young and middle-aged men; indeed, it is the most common type of cancer among males aged 15–44 years in developed countries [24]. Noticeably, over the past four decades, the incidence of this type of cancer has increased especially in wealthy, industrialized countries [25,26,27] and has started to grow in countries moving toward higher levels of development [27]. The increasing trend of TC has raised attention towards its epidemiological features and the genetic and non-genetic factors involved in the etiopathogenesis [28,29,30,31,32]. The causes of increased incidence of this type of cancer include cryptorchidism, occurring in 2–9% of boys born at term and considered to be linked to an almost 9-fold increased risk of TC, along with hypospadias; indeed, TC, cryptorchidism, and hypospadias are hallmark components of the “testicular dysgenesis syndrome” [20,33,34]. The first event in TC occurs prenatally as the presence of foci of poorly differentiated gonocytes in seminiferous tubules. Postnatally, TC development and progression is associated with allele polymorphisms of a number of transcription factors (i.e., GATA4 and GATA1), genes (i.e., PRDM14, DMRT1, SALL4, TEX14 WDR73, PMF1, CENPE, and PCNT) and receptors (i.e., CAG/GGC androgen receptor polymorphism), which are implicated in the maturation specification and differentiation of the postnatal testis [35,36,37,38]. Even though, compared to tumors in other districts, testicular cancer is still more treatable [39], both the cancer and its treatment are associated with several complications, particularly sexual dysfunction and infertility [40,41]. These, together with the expected growth in the coming decades, highlight the public health and socio-economic impacts of testicular cancers [42,43]. Nonetheless, environmental factors including heavy metals, polycyclic aromatic hydrocarbons (PAHs), polychlorinated biphenyls (PCBs), dioxins, pesticides, ultrafine particles, produced by human activities put a strain on the body’s entire defense system which struggles to metabolize and eliminate toxic compounds. Together with public health measures, evidence-based individual resilience measures are therefore necessary to mitigate health risks under conditions of environmental stress and to prevent noncommunicable diseases (NCDs); this holds true especially for workers occupationally exposed to pollutants and/or population residing in highly polluted areas. Mediterranean diet is a foremost model for nutritionally rich and balanced diets [44]. It is characterized by high intake of fruits and vegetables, rich in detoxifying and antioxidant substances, hence it is particularly rich of flavonoids. Flavonoids promote the elimination of pollutants in tissues and fluids and/or mitigate their effects through different mechanisms. In this review, the properties of flavonoids will be explored in the context of the Mediterranean diet model highlighting their role in mitigating the long-term effects of pollutants on human health with particular attention to reproductive system cancer and male infertility.

## 2. Mediterranean Diet

It is well-known, that diet can significantly influence the population’s risk profile, both at primary and secondary level of prevention. Several types of diets have been brought to public attention, but the one that has gained the most interest is no doubt the Mediterranean diet. The concept of Mediterranean diet originates from 60’s, when Ancel Keys first coined this term based on the results of an epidemiological study which showed that the populations (Italy and Greece) that faced the Mediterranean Sea presented a lower incidence of cardiovascular diseases and cancer than other populations [44]. After this famous study, other researches have corroborated these results, and the Mediterranean diet with its specific foods has been diffused all over the world as a model of a potential healthy diet. At present, the global scientific milieu highlights the Mediterranean-like diet pattern as the ideal dietary profile to maintain the health status and to reduce cancer mortality and in general the incidence of the major NCDs [45,46,47].

The Mediterranean diet is characterized by low consumption of meat, intake of vegetable oils instead of animal fats as sources of lipids, moderate amounts of red wine and significant amounts of fresh fruits and vegetables. Plant polyphenols have attracted considerable interest in the scientific communities in recent times for their health-promoting properties. In fact, many clinical studies have indicated health benefits attributable to the presence of significant quantities of these molecules, even though, in some cases, contradictory results have been reported, which highlights the need for further investigations. Therefore, recent research has sought to provide insights into the mechanisms of action of these compounds to help decipher the complex relationships between plant polyphenols and cellular homeostatic systems, including metabolic and redox balance, proteostasis, and the inflammatory response, establishing an increasingly strong molecular basis for the beneficial effects of these molecules. The underlying biochemical and molecular events include anti-inflammatory, antioxidant and anti-atherosclerotic mechanisms, as well as the epigenetic and gut-microbiota modulations pointed out by more recent studies [48]. Overall, the currently available data are providing a rationale for the possible use of natural polyphenols as nutraceuticals to counteract aging and combat many associated diseases [49]. In fact, various phytochemical compounds found in plant foods can prevent or counteract the harmful effects of environmental pollutants, through a number of mechanisms including reactive oxygen species (ROS) scavenging, chelation of toxic metals, anti-inflammation, epigenetic up-regulation of detoxifying genes or enzymes, and other [50,51,52,53,54,55,56]. Some systematic reviews found that diets rich in fish, shellfish and seafood, poultry, cereals, vegetables and fruits, low-fat dairy and skim milk were positively associated with sperm quality parameters [57,58]. 

A substantial body of research evidence from observational studies worldwide suggests that the diets’ models consistent with those promoted for the prevention of heart disease and other chronic conditions can be advantageous for male fertility as well [59]. Meanwhile, the evidence on the association between dietary patterns and male fertility is far from complete; nevertheless, several indications have emerged. The increased intake of omega-3 fatty acids, both as supplements or from foods (whether from nuts or fish) seems to positively affect spermatogenesis. Integration with antioxidants and nutrients implicated in the mono-carbon metabolism pathway (folate, vitamin B12, zinc) also seems a beneficial. In addition, a study conducted by Salas-Huetos et al., 2018 showed that supplementation of a conventional “Western” diet with walnuts, hazelnuts and almonds, present also in Mediterranean diet, improves key sperm quality parameters among healthy men of reproductive age, potentially by a reduction in sperm DNA fragmentation. These results support the potential benefits of some nutrients contained in nuts for sperm quality [60]. In a 24-week study on adult rats, Dominguez-Vias et al. [61] showed that a diet enriched with virgin olive oil, a major element of Mediterranean diet, increased the activity of dipeptidyl peptidase IV, an enzyme involved in glucose metabolism regulation as well as detoxification processes; in the testicular r tissue compared to a butter-enriched diet; this latter, contrary to olive oil enrichment, elicited a number of markers of saturated fat intake [61]. Bioactive substances in vegetable foods also deserve attention. Resveratrol (RES), abundant in grape, is a well-known cytoprotective substance with beneficial effects on diverse cell types thanks to its anti-inflammatory, anti-oxidant and anti-cancer properties. In vitro, it increased total and progressive sperm motility, restored chromatin compactness and decreased sperm lipoperoxidation, along with mitochondrial superoxide anion levels in benzopyrene-exposed spermatozoa. These biological activities deserve to be further investigated in vivo, for possible benefits in people with impaired fertility due to environmental factors [62]. A recent randomized clinical trial on healthy young males in highly polluted area of Italy has demonstrated positive effects of the Mediterranean diet and regular physical activity, on semen quality [63].

Although several studies indicate a favorable effect of the adherence to Mediterranean diet on semen parameters, few data evaluating the influence of diet on couple’s fertility are reported, including in couples attempting conception with assisted reproductive technology (ART). Thus, the impact of Mediterranean on couple fertility and ART success is a topic for further studies [64,65,66,67]. 

In addition, “organic” (i.e., cultivated based on agroecological criteria) foods, due to a higher content in bioactive compounds in comparison to conventional ones, has been suggested as an additional safeguard for counteracting the effects of environmental pollutants [68]. In this direction, the typical foods of the Mediterranean diet, when they are organically cultivated, should have a significantly reduced content of pesticides [69,70], whereas they are expected to contain higher concentrations of their natural occurring beneficial phytochemicals including polyphenols [71], flavonoids [72], carotenoids [73] and macro- and micro-nutrients such as vitamin C, iron, magnesium, phosphorus and omega-3 fatty acid [74]. Therefore, the environmentally friendly cultivation procedures endorsed by agroecology can maintain a higher content of bioactive compounds, compared to conventional foods. Indeed, several studies report that the consumption of organic foods may improve fertility, in addition to reducing other reproductive (pre-eclampsia, obesity in pregnancy) and other non-reproductive health disorders (overweight, eczema in children, some cancers, diabetes etc.). Therefore, rather than a specific effect, improved reproductive health may reflect a better health status associated with a healthier dietary style, higher intakes of nutrients, antioxidants and other bioactive compounds, as well as lower intakes of undesirable substances (i.e., nitrates and cadmium from fertilizers, pesticides, fertilizers, pollutants and their metabolites) [68,71,75,76,77,78,79,80,81]. Therefore, cultivation methods influence the potential of foods to counteract the oxidative stress and epigenetic alterations induced by environmental contaminants [51,52,53,54,55,56,57,82,83,84,85,86]. 

Communities living in polluted areas are recognized to experience a higher incidence of cancer, NCDs and male infertility. Therefore, in parallel to regulatory actions, evidence-based lifestyle intervention models should be designed in order to mitigate the impact of environmental pollutants on human health [63,87,88,89,90,91]. 

Spermatozoa are very sensitive to oxidative stress induced by environmental toxins so that the richness of antioxidants of Mediterranean diet nutrients has protective role especially on morphology, motility and sperm DNA [57,92,93,94]. Semen quality is also a potential susceptibility indicator to SARS-CoV-2 insults in polluted areas [14], and very recently it has been demonstrated that air pollution and COVID-19 could represent a possible dangerous synergy for male fertility [95]. 

## 3. Flavonoids: Mechanisms of Action in Cancer

**Figure 2:**
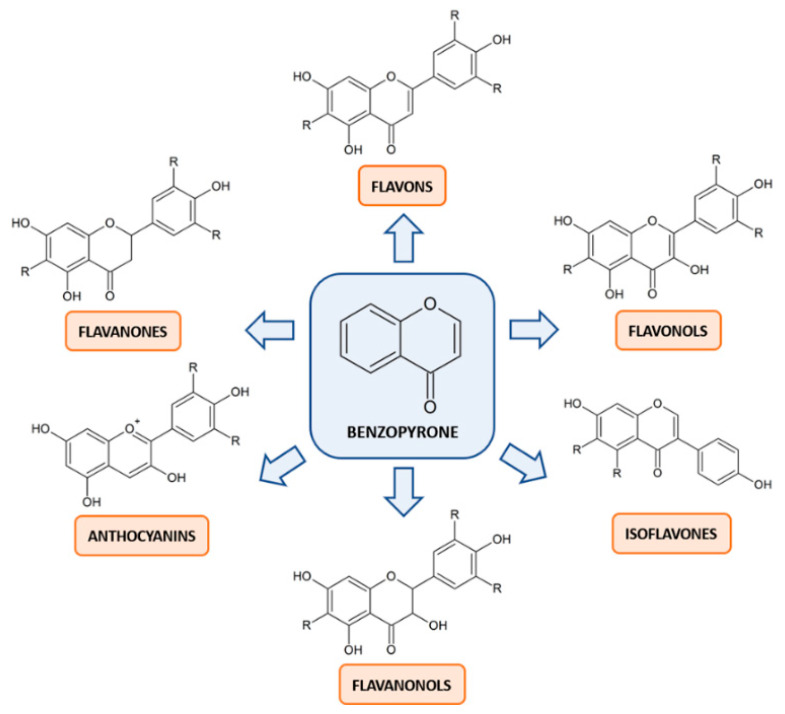
Basic structures of the six classes of flavonoids. The lead compound benzopyrone can be variously substituted to form the derivative structures of flavonols, flavones, flavanones, flavanonols, anthocyanins and isoflavones. The hydroxyl groups can be linked to monosaccharides, in case of glycosides, or methoxylated. The radicals, expressed as R, can be either methyl or methoxy groups, depending on the structure.

Flavonoids are the most prevalent and well-studied polyphenols in human diet. The common scaffold within this class of compounds is the benzo-pyrone moiety, which can be variously substituted. Based on the degree of oxidation or unsaturation, flavonoids can be classified into six major subclasses: flavonols, flavones, flavanones, flavanonols, anthocyanins and isoflavones (Figure 2). They are more frequently found as glycosides, although it is likely to find them as aglycone.

**Figure 3:**
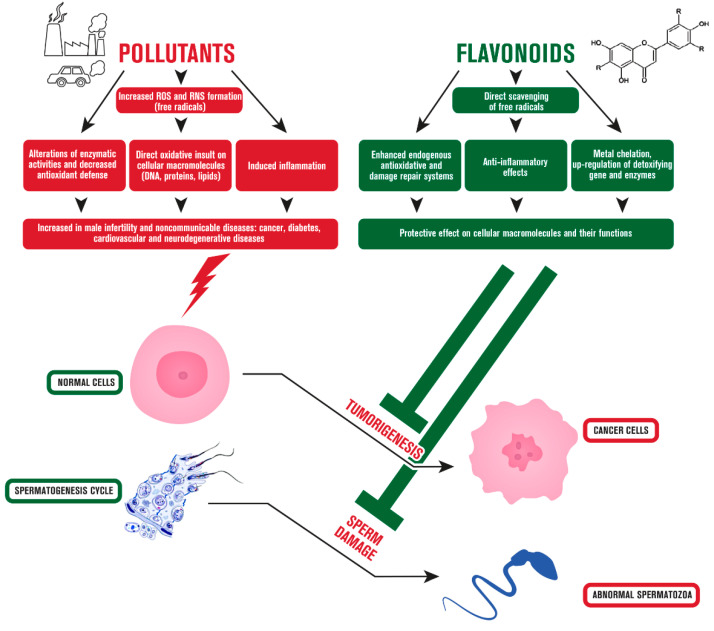
Effects of flavonoids against environmental pollutant-induced carcinogenesis and male infertility. Physiological processes of normal cells can be altered by pollutants leading to cancer and sperm cell damage. Flavonoids can protect cellular functions from environmental insults via targeting a wide plethora of mechanisms.

*Citrus* fruits, tea, and red wine are among their primary nutritional sources. Numerous studies have revealed that eating polyphenol-rich foods on a daily basis may benefit a wide range of human pathologies, including those characterized by an abnormal inflammatory and oxidant status, such as infections, autoimmune and neurodegenerative diseases, along with cancer [96,97,98,99,100,101,102]. These interesting properties are also exploited in clinical settings, mainly in the aromatherapy [103,104]. Regarding cancer, which is characterized by an uncontrolled cell proliferation and a disrupted cell cycle, leading to aberrant cells that infiltrate and metastasize to other regions of the body, flavonoid act by a wide plethora of mechanisms [105,106,107,108]. Moreover, it was documented that these compounds are able to counteract not only genetic causes of cancer development [109], but also external causes, such as pollution, smoking or radiation [110]. In particular, the ability of flavonoids to inhibit tumorigenesis was attributed primarily to their undoubted antioxidant activity, which is capable of preventing and scavenging the formation of ROS and reactive nitrogen species (RNS), known players in the toxicity of many pollutants [111]. Flavonoids also act as free radical scavengers mainly via chelating metallic ions, that consequently catalyze the generation of ROS and RNS [112,113,114]. These are the leading causes of many other conditions other than cancer, such as inflammation [115,116,117], and flavonoids target different receptors to modulate intracellular signaling pathways involved also in this process like SIRT1, nuclear factor kappa B (NF-κB), mitogen activated protein kinases (MAPKs) and cyclooxygenase-2 (COX-2) [118,119,120,121]. Flavonoids were shown to act also via immune cell regulation, suppressing chemokines and cytokines [122,123], which are known to be implied in cancer progression as well as in its spreading [124]. In this regard, flavonoids were shown to actively inhibit factors implied in metastasization of cancer via altering adhesion molecules such as metalloproteinases and other epithelial-mesenchymal transition markers [125,126,127]. Angiogenesis is another relevant process involved in cancer progression and migration, since it is crucial for the proper nourishment of the tumor microenvironment, hence the acknowledged role of flavonoid of inhibiting pivotal factors of this process, such as VEGF or EGFR expression, is one of the paths followed by these compounds to counteract cancer formation [128,129]. Among the numerous processes involved in tumorigenesis, the escape from apoptosis, which is a type of controlled cell death that is generally triggered by a number of signal transduction pathways and pro-apoptotic proteins, is acknowledged to represent one of the first characteristics acquired by cells undergoing promotion [130]. Flavonoids are able to interfere with the apoptotic machinery via hampering the activity of caspases and Bcl-2 family members [131,132]. Parallelly, flavonoids can restore also impaired autophagy in tumor cells due to the activation of beclin-1 and LC3, marker of early and late stages of autophagosome formation, respectively [133]. Moreover, the altered progression through cell cycle is another crucial element characterizing tumor development, and flavonoid were widely investigated in this field, finding their ability to modulate expression of several isoforms of cyclins, involved in each phase of cell cycle [134]. Finally, the intake of flavonoids through a varied and balanced diet, such as the Mediterranean diet, does not lead to any appreciable risk of undesirable effects, contrary to high intakes through supplements, where potential endocrine effects justify caution [135]. The effects of flavonoids against cancer development are summarized in Figure 3. 

## 4. Flavonoids: Mechanisms of Action in Male Infertility

Flavonoids have been extensively explored for the treatment of male reproductive dysfunctions. The observed favorable effects can be ascribable to a combination of biological mechanisms, for example their antioxidant, anti-inflammatory, immunostimulant, anti-apoptotic, anticarcinogenic activities [136]. Moreover, flavonoids are amphipathic molecules that can penetrate the lipid bilayer of membranes, thus providing possible protection for the entire spermatozoa and acrosome membrane. In this way, flavonoids prevent oxidative damage and guarantee the acrosome reaction of spermatozoa necessary for fertilization [137,138].

Rutin and quercetin, along with epigallocatechin, were shown to improve motility, plasma and acrosomal membrane integrity, mitochondrial activity, anti-oxidase activities, and lower intracellular ROS concentration of frozen sperm [139,140,141,142]. Among these, rutin was shown to improve the kinematic parameters of post-thawing sperm, as well as its fertilizing characteristics, prompting an increase in cleavage rates and blastocyst rate [142]. Instead, the main intercellular target of quercetin appears to be the mitochondria. Studies have demonstrated its protective and regulatory action of crucial mitochondrial processes, including electron transport chain and oxidative phosphorylation, which may affect the metabolism and performance of male gametes [140].

In male rats, quercetin reduces the endocrine and testicular alterations induced by the heavy metal cadmium [143,144]. Quercetin, apigenin, EGCG, and luteolin are also capable of increasing gene expressions of steroidogenic acute regulatory protein (StAR), cytochrome P450 11A (CYP11A), CYP17A, 17-beta-hydroxy steroid dehydrogenase (17-β-HSD) and 3-beta-hydroxy steroid dehydrogenase (3-β-HSD), useful for restoring Leydig cell function and testosterone secretion [145].

Oxidative stress is indicated as an essential contributor to apoptosis, DNA damage, lipid peroxidation, and decreased sperm motility [146]. Rutin and quercetin are able to attenuate ROS accumulation and malondialdehyde (MDA) production by improving antioxidant enzyme activity [142,147]. Moreover, quercetin eliminates the toxic effects of hydrogen peroxide (H_2_O_2_) on motility, amount of MDA and nitric oxide (NO), along with increasing viability and total antioxidant capacity (TAC) [148]. Effects of flavonoids on apoptosis have also been shown. In particular, rutin can inhibit cell apoptosis in H_2_O_2_-induced Leydig cells [149], while hesperetin lowers the number of TUNEL-positive germ cells in the testis [150]. In addition, rutin, hesperetin, and morin were noted to decrease apoptosis in testis by significantly increasing protein levels of Bcl-2 (B-cell leukemia/lymphoma 2), and decreased protein levels of BCL2-associated X (Bax) and cysteine aspartic acid-specific protease 3 (caspase 3) [149,150]. A relationship between Sertoli cells (Sc), blood-testis barrier (BTB), and flavonoids also exits. In this context, luteolin protects SCs and BTB integrity by up-regulating the expression of several antioxidant genes and ameliorating the protein expression of ZO-1, occludin, claudin-11, and Cx43 [151]. In addition, it was shown that icariin may affect spermatogenesis via regulating the claudin-11 mRNA expression in SCs [152]. Finally, investigations have established the important role that 3′,5′-cyclic adenosine monophosphate (cAMP)/ cAMP-dependent protein kinase-A (PKA) signaling pathway plays in the regulation of testosterone secretion. In this regard, another effect of quercetin, apigenin and luteolin is to inhibit cyclooxygenase-2 (COX-2)-dependent signal transduction, which has been found to enhance cAMP/PKA-dependent StAR gene expression and steroidogenesis in Leydig cells [145,153]. Overall, flavonoids show a number of biological mechanisms relevant to the protection of male fertility in in vitro and in vivo studies. The available evidence supports the design of studies on humans in areas polluted with EDC and other reproductive toxicants, in order to investigate the efficacy and safety of increasing flavonoid intake within a balanced and nutritionally rich dietary pattern, such as the Mediterranean diet.

Figure 3 summarizes the main effects of flavonoids that may counteract the pollutants affecting male.

## 5. Heavy Metals

Heavy metals are a naturally occurring part of the Earth’s crust, but anthropogenic and industrial activities have caused drastic environmental pollutions in distinct areas. The non-biodegradability of heavy metals causes them to persist in the environment. Thus, they can enter the food chain through cultivated plants, and eventually can accumulate in the human body through biomagnification. Given the toxicity of heavy metals, human health and ecosystems are threatened by their contamination. Heavy metals, however, are of utility in industrial areas such as alloying, smelting, and commercial product manufacturing. Of course, this contributes to increased exposure to heavy metals as waste from industrial processes represents a significant source of environmental contamination and subsequent accumulation in the human body. 

Almost all heavy metals are severely toxic as carcinogens. For example, arsenic, cadmium, chromium, and nickel are classified as Group 1 carcinogens by the International Agency for Research on Cancer, and are used commercially [154]. Living organisms easily come into contact with various heavy metals such as cadmium, cobalt, methylmercury, manganese, and arsenic, and it has been shown in many research studies that a strong link exists between the environmental contaminants and human health. In addition, several metals also have negative effects on the reproductive health of organisms [155,156,157,158,159,160,161,162]. Heavy metals such as lead (Pb), mercury (Hg) and cadmium (Cd) can influence the endocrine system, disrupting embryonal programming and gonadal development in utero [163]. Chromium (Cr), Cd, iron (Fe), nickel (Ni), Pb, and copper can enhance ROS [164], which can impair DNA in the male germ line and increase health risk in offspring. Some studies have shown that the risk of hypospadias and cryptorchidism is significantly increased by paternal exposure to heavy metals [165]. This study, and also another [166], proposed that the risk of hypospadias may be elevated following maternal exposure to heavy metals. In addition, a recent study provides little evidence for the association of parental exposures to heavy metals and testicular germ cell cancer in progeny except in cases of high paternal chromium exposure [164]. 

The reported results of many research suggest that increased consumption of fruits and vegetables or certain dietary supplements can substantially enhance the protection against many heavy metals. Over the past decade, dietary factors have proven to reduce the toxicity of environmental pollutants and effect prevention against adverse chronic degenerative diseases and cancer in humans, and a growing scientific body of evidence has suggested that flavonoids, thanks to their antioxidant and metal chelating properties, are involved in preventing or decreasing the damage caused by heavy metals.

### 5.1. Cadmium (Cd)

The best documented explanation for the toxicity related to Cd is oxidative stress. Cd also acts as an endocrine disruptor, particularly of the reproductive hormones [163,167]. Cd mimics the chemical state of divalent Zn; therefore, it has the potential to interfere with the site of Zn binding to DNA. Furthermore, this metal mimicking endogenous estrogen, produces an increased risk of ovarian and breast cancer and also disrupts the ovarian steroidogenic pathway, progesterone and testosterone production [168,169]. Several studies reveal that testis is a susceptible target of toxicity from cadmium. In fact, cadmium exposure can cause germ cell apoptosis and decreased daily sperm production, which may account for the decline in male fertility [170].

Regarding Cd, Xia Li et al. highlighted how flavonoids can alleviate Cd toxicity [171]. Anthocyanins have been widely applied to nutritional intervention of Cd toxicity. Flavonoids having similar structure demonstrate analogous mechanisms of action in protecting the body against Cd poisoning. These mechanisms in Cd-induced diseases mainly include elimination of ROS, reduction of lipid peroxide production, increased activity of enzymes related to oxidative stress, reduction of free Cd^2+^ content, reduction of DNA damage and cell apoptosis, inhibition of inflammation and fibrosis, and influence of metal ions in vivo. In addition, experimental studies have shown that fenugreek seed powder attenuates cadmium-induced testicular damage and hepatotoxicity in male rats [172,173]. These authors, in fact, showed that flavonoids restored the depletion of antioxidants as they antagonized the effect of Cd on antioxidant enzyme activity or increased GSH content, improving the antioxidant capacity of the body. In parallel, a flavonoid rich-extract of bergamot juice counteracted both testicular and kidney damage induced by Cd exposure in rats [174,175]. Another research asserts that flavonoids reduced the chromosomal aberration regarding to structure and number of spermatocytes due to their detoxification action that protect protein and DNA from free radicals, and hence from cancer [173]. In a similar manner, catechin hydrate exhibited protective antigenotoxic and anti-immunotoxic roles via decreasing the fragmentation of DNA and by suppressing the expression of the genes related to apoptosis [176]. Quercetin is among the most important flavonoid found in vegetables and fruits; it shows anti-inflammatory, anti-hypertensive, vasodilator, anti-obesity, anti-hypercholesterolemic, and anti-atherosclerotic effects [177], as well as for its protective against testicular carcinogenesis [178]. The protective effects of quercetin can be explained by its ability to chelate metal ions and form stable complexes given its three potential bidentate binding sites (α-hydroxycarbonyl, β-hydroxycarbonyl or catechol) [179], along with its inhibitory effects on apoptosis, cell migration, differentiation and proliferation, oxidative balance, and inflammation [180].

As regards Cd-induced-pro-oxidant action, the protective effect of dietary quercetin in testicular germ cells was documented by several research [181]. In male mice, Cd significantly decreased testicular antioxidant system, including decreases in the level of GSH, SOD and GSH-Px activity. In addition, Cd exposure resulted in increased H_2_O_2_ production and lipid peroxidation in the testes and caused germ cell apoptosis by increasing the expression of the proapoptotic proteins Bax and caspase-3 and decreased the expression of the antiapoptotic protein Bcl-XL. Notably, the administration of quercetin significantly attenuated these effects [182]. In another work, it was showed that Cd accumulated in testes, leading to oxidative stress and autophagy and that quercetin decreased cadmium toxicity by reducing oxidative stress and inhibiting autophagy [183]. In a similar way, quercetin effectively inhibited apoptosis in chicken granulosa cells exposed to Cd by regulating the inhibition of antiapoptotic protein Bcl-2 and X-linked inhibitor of apoptosis protein (XIAP) and activated caspase-3 [184]. Quercetin is highly present in onions, beans and fruits, such as apples, apricots, cherries and grapes [185]; the studies on Cd and quercetin support that a rich, varied and predominantly plant-based diet, such as the Mediterranean diet, can provide bioactive substance mitigating the effects of environmental exposures.

### 5.2. Mercury (Hg)

Plant polyphenols, which have well recognized antioxidant properties, represent very effective agents counteracting oxidative environmental stressors, including Hg. The exposure to the organic Hg form, as methylmercury (MeHg), results in the generation of reactive species that can lead to oxidative damage of macromolecules, especially DNA. Furthermore, MeHg associates with endogenous biomolecules containing thiol groups, such as glutathione. The growing occurrence of food-borne Hg is a matter of concern, given the numerous serious adverse downstream effects, ranging from kidney, cardiovascular disease and reproductive problems, whereas MeHg specifically features as a developmental neurotoxic. In addition, infertile individuals exhibited higher Hg levels in blood, hair and urine in comparison with fertile subjects. Hg exposure prompted sperm DNA damage and abnormal sperm morphology and motility. Moreover, Hg levels were correlated with a greater incidence of menstrual and hormonal dysfunction and enhanced cases of adverse reproductive outcomes [186]. Several papers report the biochemical bases for pharmacological utilization of *Fejioa sellowiana* polyphenol-rich functional food in the prevention and suppression of Hg-related health disorders [187,188]. Moreover, diverse studies have also reported the possibility of reducing mercury toxicity with flavonoid intake. Among the variety of dietary flavonoids, the beneficial role of chrysin (CR), which is found mainly in passion fruit, honey, and propolis in preventing Hg-induced alterations in both human erythrocytes and neuroblastoma cells has been investigated [189]. Finally, a number of investigations have revealed the role of olive oil polyphenols in preventing Hg toxicity [190].

### 5.3. Inorganic Arsenic (iAs)

The toxic effects of iAs on gonadal glands have been long studied through animal models, showing that iAs can be accumulated in gonadal glands and induce an inhibitory effect on gonadal development [191]. In addition, iAs affects one or more sex hormones and induces inhibition of ovarian steroidogenesis, reproductive disorders, testicular steroidogenic function, and spermatogenesis [192]. Similar toxic effects have been observed in humans. In fact, iAs has been seen to cause a variety of reproductive problems through disruption of the gonadal endocrine system. Specifically, iAs has been shown to have a detrimental effect on reproduction and development in human reducing the quantity and quality of human sperm [193]. Moreover, iAs has been linked to the onset of various forms of cancer in human, including that affecting the breast, which is the most relevant [194]. To date, no report on the direct effects of flavonoids against iAs-induced tumorigenesis is present, although it has been claimed that flavan-3-ols, flavone, flavonol, flavanone, and anthocyanidin are positively associated with urinary dimethylarsinic acid excretion in a clinical trial performed on 1037 Mexican women living in a highly polluted area [195]. This supports the hypothesis that flavonoids may hamper iAs-toxicity and hence tumorigenesis, by increasing its excretion.

## 6. Bisphenols

Endocrine disrupting chemicals (EDCs) are hormone-like agents found in the environment that induce adverse health effects by altering the endocrine system of vertebrate animals, including humans. Specifically, xenoestrogens are believed to be involved in development, reproduction, and malignant diseases by mimicking the natural hormone 17β-estradiol (E2) and interfering with endogenous endocrine regulation at specific times, such as during fetal growth. Several organochlorine pesticides-polychlorinated biphenyls (PCBs), phthalates, and bisphenol A (BPA)-used in the chemical industry have been identified as estrogenic EDCs. BPA is currently severely restricted as an EDC in the European Union, but this compound has been used from the 1950s in industrial materials, food packaging, dental sealants, and personal care products. All persons have exposure to BPA via skin, inhalation, and through the digestive system. Exposure to substances with potential for endocrine disruptive effects are continually increasing and, in recent decades, are considered the primary factor in the rising incidence of testicular cancer. Because of its metabolic and endocrine interference and its link to various human diseases including cancer, diabetes, obesity, reproductive problems, BPA is subject of intense research [196]. BPA is known to disrupt the endocrine pathways since it is a partial estrogen agonist [197] and has anti-androgenic and anti-thyroid activities [198]. For several decades, the impact of low doses of bisphenols on male reproduction has been controversial. While some investigations have reported that the low dose administration of bisphenols does not affect the vital alteration of reproductive qualities, other studies have revealed varying degrees of damage they trigger on male fertility. However, equal exposure to the same disruptor is not uniquely associated with the same phenotype of testicular dysgenesis syndrome, and this highlights the role of genetic background in establishing susceptibility to genito-urinary disorders, in general, and testicular cancer in particular [198]. Furthermore, fetal and perinatal exposure to BPA in rodents has been reported to affect the brain, mammary gland, and reproductive tract, including hormone-dependent cancer [199,200]. In addition, BPA is also capable of triggering a non-genomic action in pancreatic islets, endothelial and pituitary cells, and mammary cancer cells starting fast responses at low doses [201,202]. Flavonoids have been explored for their role against BPA toxicity. In particular, a very recent study investigated if flavonoids from *Cuscuta chinensis* (CCF) could be used as dietary supplements to reverse BPA-induced epigenetic disturbances by analyzing the molecular mechanisms linked to impairment of testicular development by BPA. The results displayed that in comparison with BPA group, CCFs were able to markedly increase the serum content of testosterone (T), estradiol (E2), the transcript levels of DNA methyltransferase 3A (Dnmt3A), Dnmt3B and that of estrogen receptor alpha (ERα). These findings suggested that CCFs could lower the levels of ERα and H19/Igf2 gene methylation by suppressing DNA methyltransferase (DNMT) expression, thus decreasing reproductive hormone and receptor levels in adult males, and thus mitigating the adverse effect of BPA on testicular development in male mice [203]. Several flavonoids, including daidzein, genistein, luteolin, chrysin, flavone, and naringenin, have been shown to exhibit anti-estrogenic activity, preventing BPA from proliferating in MCF-7 breast cancer cells and causing malignant consequences [204]. In breast cancer cell lines, ER has been found to be antagonized by naringenin, inhibiting their proliferation and supporting the role of flavonoids in preventing BPA carcinogenesis and reproductive system damage. 

Pharmacological inhibition of ERK1/2 could be considered as a target to mitigate the effects of bisphenols in testicular cells. A recent review has provided an overview to understand how oxidative stress induction may contribute to the harmful reproductive impacts induced by BPA exposure [205]. The several studies reviewed show that reproductive organs in males are more vulnerable to BPA exposure than those in females. Males may be more susceptible for several reasons. First, because there are gender differences in glutathione levels, considering its importance to the detoxification process (lower availability of glutathione in males). Also, there is greater capacity for sulfate-based detoxification in females. In addition, there is a greater inflammatory response in male reproductive organs to consider. Finally, reduced vulnerability to oxidative stress in female organs has been reported. Growing evidence shows that a wide variety of BPA doses (in vivo and in vitro or human exposure) promotes ROS generation and redox balance alteration, by leading to mitochondrial dysfunction and cell signaling pathway modulation related to oxidative stress. In addition, it should also be kept in mind that oxidative stress induced by BPA may operate in a dependent or independent manner from its endocrine and metabolic deregulatory properties and that all this may produce marked reproductive effects in prenatal, perinatal and postnatal exposure or in adulthood. Finally, the effect of BPA can be most severe when exposure is combined with other risk factors, such as a poor diet, metabolic impairments, and comorbidities [205]. The great antioxidant potential of flavonoids may also function in the body’s defense against BPA damage, including cancer.

## 7. Polycyclic Aromatic Hydrocarbons

Air pollution is known to contain various toxic substances, gases, particulates, polycyclic aromatic hydrocarbons (PAHs), toxic metals, etc. Some of them could affect the reproductive process and sperm quality by decreasing one or more sperm quality parameters i.e., sperm morphology, concentration, motility, sperm DNA damage, up to testicular cancer. The impact could be related to the concentration of pollutants and the duration of exposure [206]. PAHs, formed by pyrogenic, petrogenic and biogenic (biological) processes, are defined as persistent organic pollutants (POPs); two of them, 7, 12-dimethylbenzo[a]anthracene (DMBA) and benzo[a]pyrene (B[a]P), are among the most widely studied carcinogenic PAHs for their negative impact on the environment, human and animal health. In particular, the International Agency for Research on Cancer (IARC) defined PAHs as “probable” or “possible human carcinogens”, while benzo [a] pyrene has been reclassified in group 1 as a “human carcinogen” [207]. Scientific studies have shown that PAHs compounds undergo transformations through various metabolic reactions that occur in the body, before genuinely carcinogenic types of molecules are produced. It is precisely during these reactions that PAHs can transform into electrophilic intermediates capable of reacting with biological molecules, including DNA [6]. 

The toxicity of PAHs is related to their physical/chemical properties. PAHs are soluble in most organic solvents, are very lipophilic and have a high ability to adhere to organic material. Within 24 h, they then begin to degrade through a sequence of radical reactions, or undergo degradation by photolysis. The toxic effects occur through the formation of reactive intermediates and the activation of a particular aryl hydrocarbon receptor (AhR) [208] that regulates the expression of a series of genes. In addition to regulating the production of enzymes involved in metabolic processes, AhR also has a role in regulating the immune system, stem cells, cell differentiation and proliferation, apoptosis, carcinogenesis and drug metabolism [209]. Other evidence on infertile populations suggested a negative association between semen quality, testicular cancer, and PAH levels [210]. Moreover, male infertility can be induced by PAH by affecting sperm motility, chromatin integrity, and increased oxidative stress [211]. However, considering the human exposure at PAHs in the environment, the impact of these compounds on testicular cancer and the related mechanisms involved in toxicity could be further investigated.

As many environmental pollutants, PAHs promote the excessive production of both ROS and RNS [212]. Therefore, the maintenance of antioxidants is important in the prevention of the damage caused by environmental pollutants, such as PAHs. 

Matzkin et al. [213], reported how anti-inflammatory and antioxidant compounds could exert a beneficial role in the improvement of physio-pathological state of the aged male gonad [214]. Similarly, a dimer of epicatechin from the endophytic fungus *Curvularia australiensis* reverted the tumorigenic effect of B[a]P exposure in female rats on induced cervical cancer by reducing oxidative stress markers and pro-inflammatory ones, thus ameliorating lesion histopathology [215]. Other authors [216] investigated the molecular mechanisms associated with the cytoprotective effect of quercetin and its metabolites against benzo[a]pyrene (B[a]P), suggesting the involvement of other mechanisms beyond the antioxidant activity. In particular, the reduction of B[a]P-induced cytotoxicity by quercetin and isorhamnetin occurs through a decrease of BPDE-DNA adducts and intracellular B[a]P and its metabolite, increasing the xenobiotic detoxification metabolism. Along the same line, an anthocyanin-rich pool from grape proved to enhance detoxification capacity of MCF-7 cells, blocking carcinogen-DNA adduct formation as well as ROS [217]. Moreover, eupatorin-5-methyl ether, a flavonoid present in several medicinal plants, hampered B[a]P stimulating effects in MCF-7 cancer cells, via inducing p21, JNK and p-JNK expression [218].

## 8. Dioxins

Dioxins are POPs that are largely produced by industrial combustion processes such as waste incineration, the manufacturing of chlorophenols and chlorophenoxy herbicides, metal processing, and free chlorine bleaching of paper pulp. Dioxins and similar compounds all act by interacting with AhR, albeit with different potency. The cumulative effects of dioxins are assessed using as parameter the AhR-binding potency of each congener relative to that of 2,3,7,8-tetrachlorodibenzo-p-dioxin (TCDD), the most known member of the dioxin-like class [219]. The role of dioxins on the onset of several types of cancer and the relative increase of mortality has been widely established by the numerous cohort and case-control studies [220]. Moreover, human exposure to dioxins has been linked to decreased semen quality, menorrhagia, and hampered male characteristic development more than female counterpart [221]. As already mentioned, these noxious effects are claimed to be ascribed to the interaction of TCDD to the AhR, protein which translocates into the nucleus to promote the transcription of several factors involved in survival, invasive and migrative potential of cancer cells [222]. Moreover, AhR is implied in reproductive function in both sexes, being widely spread in ovaries and testes. Indeed, it is known that testicular functions (i.e., spermatogenesis and sperm motility), and hence male fertility, are affected by activation of AHR induced by environmental toxicants [223]. Therefore, AhR has been studied as an interesting target of pollutant-induced cancers and, specifically, natural products, among which flavonoids, have seized the attention of the scientific community in these regards [224]. In particular, the isoflavone genistein from soy was showed to hamper TCDD-dependent downregulation and methylation of BRCA-1, whose alteration is known to play a pivotal role in the onset of breast cancer, along with decreasing levels of DNMT-1 and cyclin D1 in MCF-7 cells, proving the role of genistein in counteracting ER (estrogen receptor) α-positive breast cancer cells via antagonizing AhR [225]. These effects elicited by genistein were seen by the same group also in triple-negative breast cancer cells [226]. The flavanone hesperetin, mainly found in *Citrus* fruits, was proved to inhibit AhR translocation induced by TCDD exposure in MCF-7 cells, along with reducing gene expression of different cytochrome P450 isoforms and other xenobiotic metabolizing enzymes [227]. Kaempferol, a tetrahydroxyflavone present in many plant species, inhibited the TCDD-induced expression of both phase I and phase II drug-metabolizing enzymes (i.e., CYP1A1, NQO1, HO-1, GSTP1) in hepatocarcinoma HepG2 cells, targeting AhR and Nrf2 pathways, and its combination with luteolin proved to bring even stronger effects [228]. Kaempferol and luteolin, along with fisetin, apigenin and naringenin hampered the TCDD-induced up-regulation of CYP1A1 and UGT1A1 in colon carcinoma Caco2 cells, although other flavonoids, such as quercetin and morin, increased the effect of TCDD. These effects were explained by the different binding modes of these compounds to AhR, since specific residues interacted with antagonists (i.e., apigenin) and others with agonists (i.e., quercetin), while other residues were in common with both species [229]. These reports highlight the ability of dietary flavonoids to counteract AhR activation mediated by dioxin-like compounds like TCDD. Nevertheless, other members of this family proved to possess TCDD-like effects on AhR, thus caution and more evidence are required for a risk-to-benefit assessment of nutraceutical or pharmacological uses.

## 9. Phthalates

Phthalates, or phthalic acid diesters, are often employed to improve the flexibility, pliability, and elasticity of otherwise hard polymers. Given their chemo-physical characteristics, they are used in a wide range of industrial and consumer items, including toys, paints, adhesives, personal-care products, and a variety of medical equipment. However, due to their EDC properties, several phthalates in EUROPE are undergoing severe restrictions. In particular, the main phthalates with EDC properties present in food contact materials are di-butylphthalate (DBP), butyl-benzyl-phthalate (BBP), bis(2-ethylhexyl) phthalate (DEHP), di-isononylphthalate (DINP) [230]. DEHP and other phthalates with EDC properties have been linked to changes in puberty, development of testicular dysgenesis syndrome, cancer, and reproductive problems in both men and women. This because phthalates can influence the release of hypothalamic, pituitary, and peripheral hormones, along with interfere with nuclear and membrane receptors, intracellular signaling pathways, and affect gene expression involved with reproduction [231]. In particular, it has been shown that a chemokine secreted by immune cells, namely Chemokine (C-C motif) ligand 5 (CCL5) and also known as RANTES, is involved in the crosstalk to maintain the correct environment for optimal spermatogenesis [232]. Interestingly, the link between toxicity by environmental pollutants, such as dioxins, and impairment of male fertility has been ascribed also to a decrease of CCL5 expression, which experimentally induced a transitory decline in sperm reserves in the testes of TCDD-exposed rats [233]. In this field, a flavonoid present mainly in *Citrus* fruits, namely didymin, reverted the cancerous effects induced by several phthalate esters (i.e., BBP, DBP, DEHP) in an in vitro experimental model targeting the CCL5 pathway [234]. In particular, it was shown that MDA-MB-231 breast cancer cells co-cultured with monocyte-derived dendritic cells and exposed to phthalate esters displayed higher rates of progression, invasion and migration due to CCL5 release. Didymin was able to deplete its secretion, thus hampering the metastatic and proliferative activities of cancer cells and proving its role in cancer prevention via targeting CCL5 pathway, and possibly protect against phthalate-induced infertility. Notably, flavonoid consumption, specifically anthocyanidins and flavan-3-ols, has been also proved to be positively associated with reduced breast cancer-risk in a population-based case-control study (233 cases and 221 controls) performed in a highly polluted area of Mexico [235]. 

## 10. Conclusions

The unceasing development of anthropic activities brings consequently an ever-increasing level of air and soil pollutants, known to hamper human, animal and plant health. In order to increase one self’s defenses, secondary metabolites from edible plants may represent valuable allies, when assumed within a varied and well-balanced diet, such as the internationally recognized Mediterranean Diet model. In these regards, flavonoids are acknowledged for their beneficial effects on human health, including the mitigation of health risks from environmental pollutants. As here reviewed, the impairment of male fertility and gonadal development, as well as progression of cancers of reproductive systems of both sexes, due to the exposure of organic and inorganic pollutants, seemed to be counteracted by flavonoids in several pre-clinical and clinical studies. While the available evidence is promising, further studies are needed to definitively assert the role of flavonoids for the protection of both fertility and related cancers caused by the exposure to environmental pollutants. This will allow to design novel evidence-based dietary strategies to defend those subjects’ health who live or work in highly polluted areas. 

## Data Availability

Not applicable.
